# Union of a Wound in the Intestine, Returned without Suture

**Published:** 1849-03

**Authors:** B. J. Pennock


*Union of a wound in the Intestine, returned without suture.* By B. J. Pennock, M.D. Communicated by W. H. Tingley, M.D.

The subject of this communication was a man of temperate and frugal habits, aged 35 years. On a day in July last, a negro in a state of intoxication, came to his house, and conducted himself in so violent a manner as seriously to intimidate the female members of his family. The man having given proper warning, proceeded to enforce his rights; as he raised a billet of wood to strike the negro, the latter fell on one knee, drew from his pocket a clasp knife, and thrust it into the abdomen of the unfortunate man. W. H. T.

" The wound in the abdomen was three inches in length, commencing in the *linea alba* three inches below the umbilicus, and extending to the right obliquely upwards and outwards; about four feet of small intestine (the ileum) with its mesentery protruded through the wound. There was much gaseous distension of the bowels, excepting, in one place, where there was a transverse wound, three-fourths of an inch in length, through which protruded a lumbricoid worm. The protruded parts were firmly grasped by the lips of the wound, causing much pain and sickness, with frequent attempts to vomit; each effort forcing out additional portions of the intestine, with considerable quantities of blood. The ' shock' was so great, and the symptoms so severe, as to induce the belief that the man would not long survive, and hence the bowel was returned without suture. Twice was I foiled in my efforts to return it, in consequence of the efforts to vomit forcing it out with much violence. The next day there was pain, tenderness, hardness and swelling of the belly. This condition, somewhat augmented, continued throughout the subsequent stages of the case, though not with such violence as to proclaim the most intense form of peritoneal inflammation. The fact is, the man never completely rallied from the ' shock,' but continued sick, and vomited many times daily until his death. Probably this prevented the violent reaction which otherwise might have been anticipated. We tried blood-letting in the early stages of the case, but found the loss of a few ounces produced so much prostration and sinking of the pulse, that we were obliged to discontinue it. After this, purgative and anodyne enemata, and blisters constituted the treatment. I may here remark, as evidence of the violence sustained by the brain and nervous system, that in two or three days the patient's mind bore traces of aberration, which increased to constant delirium before his decease. On the third day the colon was emptied by a mild enema ; and on the fifth day, there was a copious bilious discharge, which, of course, had passed the wounded part of the bowel. The bowels were easily moved at all times, by injections, and the urinary discharge was so copious as to lead to the prediction that there could be no serious effusion in the abdominal cavity, which proved to be correct. He died on the eleventh day after the reception of the wound."

"*Post-mortem.*—We had much difficulty in opening the abdomen, because of the extensive adhesions of the viscera to its walls. The convolutions of the intestines were firmly agglutinated together, as also were the folds of the mesentery. The omentum *majus* was strongly adherent to the abdominal parietes, and contained in a sac formed by its adhesions with the bowels; on separating the surfaces, we found in several places, small sacs containing purulent matter. The *cæcum* and *ascending colon* seemed imbedded in adhesions to the neighbouring parts, and were very tender, though not much discoloured. The *transverse* and *descending* colon shared in the adhesions, although they were not so dense as at its initial portion. There was no inflammation of the peritoneal covering of the stomach and liver. There was no coating of coagulable lymph, excepting in one or two spots ; neither was there much injection of the peritoneum. I was very careful in the dissection not to injure the intestines, as to allow their contents to escape, and I was successful. There were no traces of *fæcal extravasation* discovered on the most careful examination; and what much surprises us, we could not, after a tedious search, discover such appearances as satisfied us that we had found the situation of the wound in the bowel, notwithstanding we took out and examined every part of the small intestines, internally and externally; from this fact we might suppose that the colon was the injured organ; but in the bowel, which protruded at the site of the wound, there were no longitudinal or transverse bands which anatomically designate this organ. The lumbricoid worm was not mistaken for the *vermiform appendix*, for I removed the former at the time of the injury.

About five ounces of blood, partly coagulated, were found in the pelvis, the presence of which, during life, was not indicated by "fluctuation," "percussion," constipation of the bowels, or suppression of urine; it probably came from a branch of the epigastric artery."

